# Prevention, Recognition, and Management of Anastomotic Leakage and Pelvic Sepsis After Rectal Cancer Surgery: A Structured Narrative Review

**DOI:** 10.3390/jcm15166427

**Published:** 2026-08-20

**Authors:** Koji Morohara, Tsunekazu Hanai, Kenji Oshima, Yui Kitagawa, Shiho Okada, Kosuke Mochizuki, Kazuma Horiguchi, Hiroki Tani, Yoshiki Kunimura, Takashi Imanaka, Takahiro Tashiro, Yuka Kondo, Hidetoshi Nagata, Hiroyuki Kato, Zenichi Morise, Akihiko Horiguchi, Hidetoshi Katsuno

**Affiliations:** Department of Gastroenterological Surgery, School of Medicine, Fujita Health University, Bantane Hospital, 3-6-10 Otobashi, Nakagawa-ku, Nagoya 454-8509, Aichi, Japan

**Keywords:** rectal cancer, minimally invasive surgery, anastomotic leakage, pelvic sepsis, robot-assisted surgery, indocyanine green, endoluminal vacuum therapy, stoma-free survival

## Abstract

Anastomotic leakage (AL) and pelvic sepsis remain important causes of morbidity after restorative rectal cancer surgery, affecting mortality, stoma-free survival, bowel function, and oncologic treatment. Reported rates after low anterior resection are approximately 5–20%. We conducted a structured narrative review of PubMed/MEDLINE and the Cochrane Library through 20 July 2026, focusing on prevention, recognition, source control, and long-term recovery after low pelvic reconstruction. Most biological requirements for healing and principles of source control are approach-agnostic, whereas open, laparoscopic, and robotic surgeries create different technical conditions for pelvic exposure, stapler trajectory, articulation, tactile feedback, conversion, and fluorescence imaging. This review therefore integrates approach-specific technical constraints with post-discharge recognition, anatomy-based source control, and patient-centered long-term outcomes rather than treating these domains separately. Among preventive measures, combined mechanical bowel preparation and oral antibiotics has the strongest support in elective, non-obstructed patients; indocyanine green fluorescence is a useful adjunct when perfusion is uncertain but does not replace assessment of tension or mechanical integrity. C-reactive protein is mainly useful for ruling out major complications, whereas contrast-enhanced computed tomography remains first-line imaging and pelvic magnetic resonance imaging is best reserved for selected small defects, chronic sinuses, or complex pelvic sepsis. Management should be driven first by physiology and then by leak timing and location, defect size, conduit viability, diversion status, and cavity drainability. Durable success includes sepsis control, anatomical healing, stoma reversal when feasible, and acceptable long-term function.

## 1. Introduction

Anastomotic leakage (AL) after anterior resection ranges from a radiologic defect in an otherwise well patient to pelvic abscess, generalized peritonitis, or septic shock. This breadth partly explains why reported rates vary so widely. Studies differ in their definitions, surveillance policies, anastomotic levels, use of neoadjuvant treatment, and case mix. For low and ultralow anastomoses, clinically relevant AL is generally reported in 5–20% of patients [1,2,3,4,5,6,7]. The problem often persists long after discharge. Repeated interventions, chronic presacral sinus, anastomotic stricture, delayed or failed stoma reversal, impaired bowel function, and poorer quality of life are all well-recognized sequelae [8,9,10,11,12,13,14].

Laparoscopic surgery and robot-assisted surgery (RAS) are established approaches to rectal cancer, but randomized trials have not shown that either platform abolishes the risk of AL [15,16,17,18,19,20,21]. What differs is the setting in which the distal rectum is divided, and the anastomosis is fashioned. Open surgery offers palpation and manual exposure, yet access to the deep pelvis may still be limited. Laparoscopy provides magnification but constrains instrument and stapler angles. Robotics improves articulation and image stability, although the surgeon loses tactile feedback. These practical differences can affect traction, distal transection, stapler firing number, perfusion assessment, and the ability to achieve a tension-free reconstruction [4,5,6,7,15,16,17,18,19,20,21]. Integrated fluorescence imaging and earlier discharge after minimally invasive surgery add further considerations, both in the operating room and during postoperative surveillance [22,23,24].

Previous reviews have addressed colorectal complication prevention broadly or focused on endoscopic treatment of postoperative leaks and fistulas [25,26]. The distinctive contribution of this review is to connect four decisions that are often discussed separately: the approach-specific technical constraints of low pelvic reconstruction, recognition of leakage after short-stay recovery, anatomy-based source control, and restoration of durable bowel continuity. We therefore organize the evidence around the clinical course from reconstruction to recovery, asking which technical failures are preventable, how delayed presentation should be recognized, how physiology and anatomy should guide escalation, and how acute decisions affect stoma-free survival and bowel function. Accordingly, the minimally invasive perspective here is not that laparoscopy or robotics changes the biology of leakage, but that these approaches modify technical failure modes and intraoperative opportunities for prevention [15,16,17,18,19,20,21,22,23], while short-stay recovery shifts part of early recognition beyond the hospital [24].

## 2. Literature Search and Scope

We searched PubMed/MEDLINE (National Library of Medicine, Bethesda, MD, USA; https://pubmed.ncbi.nlm.nih.gov/; accessed on 20 July 2026) and the Cochrane Library (Cochrane, London, UK; https://www.cochranelibrary.com/; accessed on 20 July 2026) for publications from 1 January 2010 to 20 July 2026. Earlier landmark reports were retained when they introduced definitions or evidence that continues to shape current practice. Search terms were used in combinations and included “rectal cancer,” “low anterior resection,” “anastomotic leak,” “pelvic sepsis,” “laparoscopic,” “robotic,” “conversion,” “learning curve,” “fluorescence angiography,” “diverting stoma,” “bowel preparation,” “oral antibiotics,” “obstruction,” “intraoperative colonic irrigation,” “stapler firings,” “dog-ear,” “air leak test,” “reinforcement suture,” “transanal drainage,” “C-reactive protein,” “procalcitonin,” “computed tomography,” “rectal contrast,” “magnetic resonance imaging,” “endoscopic vacuum therapy,” “anastomotic salvage,” “chronic presacral sinus,” “anastomotic sinus,” “low anterior resection syndrome,” and “stoma reversal.” We also screened the reference lists of relevant guidelines, randomized trials, systematic reviews, and large cohort studies.

We included evidence involving adults undergoing restorative rectal resection and gave priority to rectal cancer, particularly low colorectal and coloanal anastomoses. Guidelines, randomized controlled trials, systematic reviews, meta-analyses, and large multicenter cohorts were considered first; smaller technical series were used when higher-level evidence was unavailable, particularly for salvage procedures and complex chronic pelvic sepsis. Studies from broader colorectal populations were included only when their findings could reasonably be applied to rectal anastomotic construction, diagnosis, or source control. Eligibility was assessed against the stated scope and evidence hierarchy. One author (K.M.) performed the initial screening and eligibility assessment, and a second author (H.K.) independently reviewed potentially eligible publications. Any discrepancies were resolved through discussion and, when necessary, consultation with a senior author (T.H.). For each included report, we considered the study population, operative approach, anastomotic level, leakage definition and grade, intervention, comparator, quantitative outcome, and major methodological limitations.

Because the available studies differed substantially in leakage definitions, anatomical severity, treatment selection, and criteria for success, a de novo meta-analysis was not appropriate. We therefore used a structured narrative synthesis, separating randomized evidence from observational findings and interpreting effect estimates within their original clinical context. Consistent findings were given greater practical weight than isolated or noncomparative signals, while conflicting or low-certainty evidence is identified explicitly. Numerical values in the tables indicate the likely scale and uncertainty of outcomes and should not be read as a ranking of non-randomized treatment strategies.

No instruments, devices, or statistical software packages were used to generate data or perform analyses for this narrative review. OpenAI ChatGPT (GPT-5.6 Thinking; OpenAI, San Francisco, CA, USA; https://chatgpt.com/; accessed on 20 July 2026) was used to assist with English-language editing, organization, and clarity. It did not independently select studies, extract outcomes, interpret evidence, or verify references. Every AI-assisted passage was reviewed by the authors, checked against the cited source, and revised where necessary. The authors remain fully responsible for the originality, accuracy, and integrity of the manuscript.

## 3. Clinical Context, Burden, and Risk Stratification

The International Study Group of Rectal Cancer (ISREC) defined AL as loss of intestinal-wall integrity at a colorectal or coloanal anastomosis that creates communication between the intra- and extraluminal compartments; a pelvic abscess adjacent to the anastomosis is included [1]. ISREC grades leakage according to the treatment it prompts: Grade A requires no active intervention, Grade B requires treatment without relaparotomy, and Grade C requires reoperation [1,2]. Because treatment can escalate over time, grade alone is insufficient for management. Clinically useful description also requires anastomotic height, defect size and circumference, cavity size, intraperitoneal or extraperitoneal location, tissue viability, and diversion status [24,27,28,29].

Reported incidence is strongly influenced by surveillance and case mix. Routine contrast studies detect more asymptomatic defects, whereas symptom-triggered investigation identifies fewer total events but a larger proportion of clinically relevant leaks. After low anterior resection for rectal cancer, rates of approximately 5–20% have been reported, with higher risk for very low colorectal or coloanal anastomoses [4,5,6,7]. An “expected leak rate” is therefore interpretable only when the leakage definition, anastomotic level, surveillance policy, and use of neoadjuvant therapy are stated.

The consequences extend beyond acute infection. AL may require antibiotics, drainage, diversion, intensive care, reoperation, or readmission and is associated with increased mortality when reintervention is required [27,28,29,30,31]. Chronic presacral sinus, stricture, reduced neorectal compliance, failed stoma reversal, and LARS may persist long after apparent control of sepsis [8,9,10,11,12,13,14]. Associations with local recurrence and poorer survival have also been reported, although confounding by tumor stage, comorbidity, leak severity, and delayed adjuvant treatment limits causal interpretation [32,33,34,35]. The clinically important endpoint is therefore not simply closure of the acute defect but recovery without chronic sepsis, permanent diversion, or unacceptable functional loss.

Risk is cumulative rather than binary. Repeatedly reported patient-related factors include male sex, visceral obesity, malnutrition or hypoalbuminemia, sarcopenia, diabetes, smoking, heavy alcohol use, renal or cardiopulmonary disease, frailty, and higher American Society of Anesthesiologists status [4,5,6,7]. Tumor- and treatment-related risk increases with a very low anastomosis, neoadjuvant radiotherapy or chemoradiotherapy, obstruction, and bulky disease that makes pelvic dissection more demanding [4,5,6,7]. Modifiable factors should be corrected when oncologically appropriate; fixed factors should lower the threshold for senior involvement, selective diversion, and intensified surveillance rather than automatically precluding restorative surgery.

Risk assessment should be updated intraoperatively because technical findings can outweigh a favorable preoperative profile. Inadequate reach, questionable perfusion [22,23], repeated or oblique stapling, uninspected staple-line corners [36,37,38], tissue injury, contamination, major blood loss, an incomplete donut, or a positive leak test [39,40] should trigger problem-specific correction rather than acceptance of a compromised anastomosis. Options include further mobilization, more proximal transection, reconstruction, selective reinforcement, diversion, or abandonment of unsafe reconstruction. Table 1 summarizes which risk factors are modifiable and how they should change operative planning; the evidence levels are a pragmatic qualitative synthesis rather than a formal GRADE assessment.

## 4. Surgical Approach as a Technical Modifier

Healing depends on perfusion, absence of tension, mechanical integrity, and control of contamination regardless of platform; the operative approach modifies how reliably those conditions can be achieved. Open surgery provides palpation and flexible instrument exchange but may still be difficult in a deep, narrow pelvis. Laparoscopy offers magnification but can constrain stapler angles and create tissue torque or multiple firings. Robotics improves articulation, image stability, and ergonomics but removes tactile feedback and introduces docking, arm-positioning, and team-learning considerations. Randomized trials have not shown a consistent platform-specific reduction in AL [15,16,17,18,19,20,21]. The practical implication is therefore to judge each platform by its failure modes, not by assumed superiority, and to change traction, port position, stapler entry, mobilization, or approach before accepting an unsafe transection. Table 2 summarizes these platform-specific constraints and preventive responses.

In minimally invasive practice, prevention therefore begins before a stapler is fired. A poor distal angle, excessive traction, repeated attempts to obtain reach, or a need for multiple firings should be treated as signals to modify the setup rather than as inconveniences to be accepted. Additional mobilization, repositioning of ports or robotic arms, a different stapler entry trajectory, or conversion when adequate exposure cannot be obtained are preventive decisions because they address the mechanism before the anastomosis is completed. Fluorescence assessment can add visual information about perfusion, but it cannot compensate for tension, poor geometry, or incomplete mechanical integrity [22,23,36,37,38,39,40]. This framing is particularly relevant to minimally invasive surgery, where enhanced visualization coexists with restricted instrument trajectories and reduced or absent tactile feedback [15,16,17,18,19,20,21,22,23].

Minimally invasive recovery also changes the surveillance context. Earlier discharge means that some low pelvic leaks first become apparent at home, while diversion may blunt feculent drainage or generalized peritonitis [24,27,28,43]. High-risk patients should leave hospital with named warning symptoms, a direct route back to the colorectal team, and a low threshold for reassessment during the first two postoperative weeks. Poor appetite, fatigue, urinary symptoms, pelvic pressure, ileus, or low-grade fever may be more informative than wound appearance or normal drain output.

When reintervention is required, physiology and source control take precedence over platform preference. In a stable patient with localized contamination, laparoscopy can permit lavage, targeted drainage, diversion, and occasionally repair; robot-assisted reintervention may be feasible in selected experienced centers but is supported mainly by noncomparative reports [28,29,42,44,45]. In instability, generalized contamination, or inadequate exposure, conversion to open surgery is appropriate and should not be regarded as failure. The clinically relevant question is whether the chosen route can achieve definitive source control without delay.

## 5. Prevention: Prioritize Correctable Mechanisms

### 5.1. Preoperative Preparation and High-Risk Situations

Preoperative prevention should target reversible risk without inappropriate delay of cancer treatment. Nutritional depletion, anemia, hyperglycemia, smoking, alcohol misuse, dehydration, renal dysfunction, and cardiopulmonary instability should be addressed when feasible [4,5,6,7,43]. Direct evidence that any single optimization measure prevents AL is limited, so the main value of prehabilitation is cumulative risk reduction and better selection for diversion rather than a guaranteed effect on leakage.

Among preoperative interventions, combined mechanical bowel preparation (MBP) and oral antibiotics has the strongest support for elective, non-obstructed colorectal resection [41,43,46]. MBP alone has not consistently reduced AL or surgical-site infection, and evidence for oral antibiotics without MBP is less mature. The quantitative signal favoring the combined regimen is summarized in Table 3; observational designs and differences in antibiotic protocols, renal function, frailty, and tolerance limit attribution of benefit to any single component.

Obstruction should be treated as a different clinical state rather than an exception to routine bowel preparation. In a dilated, edematous, or unprepared colon, cathartic MBP may be ineffective or unsafe; decompression, proximal-colon viability and caliber, contamination, and mismatch should be assessed before low pelvic reconstruction [4,5,6,7,41]. Selected preoperative diversion or bridge-to-surgery decompression may convert an emergency into a controlled reconstruction. If continuity is restored at the same operation, decompression or on-table lavage cannot compensate for ischemia, severe edema, contamination, or poor geometry; staged reconstruction or an end stoma may be safer [27,28,29,47].

### 5.2. Anastomotic Construction, Perfusion, and Protection

Construction remains the central modifiable intraoperative determinant. The proximal colon should reach without tension, torsion, or major caliber mismatch, and both ends should be well perfused. Distal transection should be perpendicular and use the fewest feasible cartridges; the association between multiple firings and higher AL is summarized in Table 3 [36]. Rather than accepting repeated oblique firings as an unavoidable consequence of a narrow pelvis, the surgeon should change port position, stapler trajectory, mobilization, or approach.

The completed anastomosis requires deliberate verification. Residual staple-line corners should be incorporated, removed, or carefully inspected; donut completeness and an air or endoscopic integrity test should be checked [37,38,39,40]. An incomplete donut, positive leak test, or doubtful perfusion is a finding that demands corrective action, not simply proximal diversion. Table 4 condenses the recommended responses, including when reconstruction is preferable to local reinforcement.

**Table 3:** Selected quantitative evidence relevant to prevention, early diagnosis, and clinical impact.

Intervention or Test	Population/Evidence	Key Quantitative Finding	Interpretation
Combined MBP + oral antibiotics	Guideline and large observational cohorts [41,46]	AL approximately 2.8% with combined preparation vs. 5.7% without; adjusted OR 0.57 in another cohort.	Preferred elective regimen; modify or omit cathartic preparation in obstruction.
Rectal stapler firings	Meta-analysis of laparoscopic rectal surgery [36]	AL 3.5% after one firing vs. 6.7% after two; OR 2.44 (95% CI 1.34–4.42).	Avoid repeated or oblique firings when the strategy can be changed.
ICG fluorescence angiography	EssentiAL randomized trial in minimally invasive rectal cancer surgery [23]	All-grade AL: 7.6% with ICG vs. 11.8% control; RR 0.64.	Useful perfusion adjunct; randomized evidence remains mixed.
Diverting stoma	Meta-analysis of randomized trials after low anterior resection [48]	Clinically relevant AL: approximately 6.3% diverted vs. 18.3% non-diverted.	Reduces clinical consequences; does not ensure anatomical healing.
CRP, POD 3–5	Meta-analyses of colorectal surgery [49,50,51]	Approximate rule-out cutoffs: POD 3, 148–172 mg/L; POD 4, 123–124 mg/L; POD 5, 115–144 mg/L. NPV near 97% in the 2014 meta-analysis [49].	Rule-out aid; interpret trends in clinical context.
CT with rectal contrast	Cohort study of suspected colorectal AL [52]	Sensitivity/specificity 78%/94% with rectal contrast vs. 47%/88% without; 93%/97% when contrast reached the anastomosis.	Useful when stable and safe; an early negative CT does not exclude AL.
Pelvic MRI	Preliminary study of 23 suspected rectal AL cases [53]	Sensitivity 94.4%; specificity 80%; accuracy for anastomoses <5 cm was 93.7%.	Problem-solving tool; not a replacement for acute first-line CT.
90-day mortality	Swedish population cohort, 6948 anterior resections [31]	90-day mortality was 3.9% with AL versus 1.5% without AL; adjusted OR 2.64 (95% CI 1.65–4.22).	Reintervention-requiring AL carries the greatest mortality burden.

Abbreviations: AL, anastomotic leakage; CRP, C-reactive protein; CT, computed tomography; ICG, indocyanine green; MBP, mechanical bowel preparation; MRI, magnetic resonance imaging; NPV, negative predictive value; OR, odds ratio; POD, postoperative day; RR, relative risk.

Indocyanine green (ICG) fluorescence angiography is best interpreted as an adjunct for uncertain perfusion rather than universal protection. Randomized evidence is mixed: EssentiAL showed a reduction in all-grade AL [23], whereas IntAct did not significantly reduce its primary clinical AL outcome [22], and ICG-COLORAL was neutral in a population that excluded low anterior resection [54]. This inconsistency argues for selective use when the fluorescence result can change the transection level or prompt reassessment. Dose, timing, camera distance, hemodynamics, and visual interpretation remain unstandardized; fluorescence cannot assess tension or mechanical integrity, and reinforcement or diversion does not correct ischemia.

**Figure 1:**
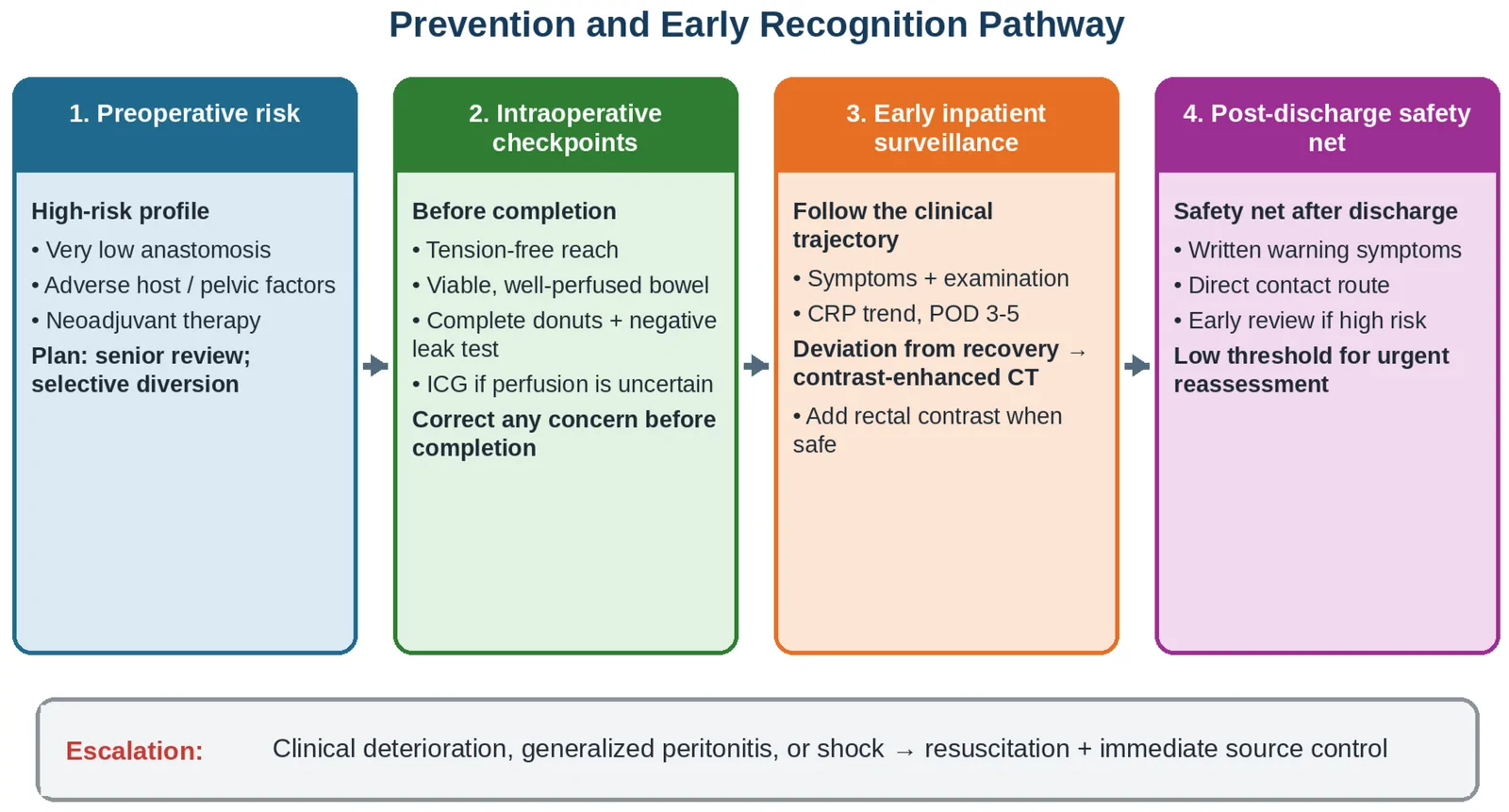
**Prevention-to-recognition pathway for AL after rectal cancer surgery.** Examples of early warning features include fever, tachycardia, pelvic pain, ileus, urinary symptoms, abnormal drain output, poor appetite, fatigue, pelvic pressure, and low-grade fever. Approximate CRP rule-out ranges from pooled colorectal data are POD 3, 148–172 mg/L; POD 4, 123–124 mg/L; and POD 5, 115–144 mg/L [49,50]. These values should be interpreted in clinical context and do not replace clinical judgment. High-risk patients require a low threshold for reassessment during the first 2 postoperative weeks. Abbreviations: CT, computed tomography; ICG, indocyanine green; POD, postoperative day. The colors distinguish pathway stages rather than quantitative risk levels.

Protective adjuncts have unequal evidentiary support. A diverting stoma has the strongest evidence for reducing the clinical consequences and reoperation burden of a low pelvic leak, although it does not guarantee anatomical healing and carries dehydration, renal, skin, obstruction, and non-reversal risks [11,13,30,48]. Transanal drainage tubes may reduce leakage in selected patients, but heterogeneous devices and protocols make the evidence less robust and they should not substitute for diversion when overall risk is high [55,56]. Routine prophylactic pelvic drainage is not supported by randomized evidence; selective use may be reasonable after contamination or a technically difficult operation, but drain output cannot exclude a compartmentalized pelvic collection [57]. Table 3 summarizes the comparative strength and quantitative scale of these preventive signals. The prevention-to-recognition pathway from preparation to early diagnostic work-up is summarized in Figure 1.

**Table 4:** Intraoperative technical findings and recommended responses before completing a low pelvic anastomosis.

Finding	Interpretation	Recommended Response	What Should Not Be Accepted
Multiple or oblique rectal stapler firings	Intersecting staple lines; restricted pelvic access; higher AL signal with multiple firings [36].	Change port/entry, traction, mobilization, or strategy; aim for perpendicular transection with the fewest feasible cartridges.	Complex staple line accepted for convenience.
One or both rectal staple-line corners outside the circular excision	Residual dog-ear; mechanically vulnerable segment [37,38].	Inspect the circumference ± endoscopy; air-leak test; reconstruct if geometry or perfusion is uncertain; reinforce only a small viable defect [37,38,39].	Uninspected corner or ischemic tissue.
Incomplete donut	Possible incomplete full-thickness tissue capture [40].	Inspect circumference + air-leak test; reconstruct if capture is uncertain; repair only a small viable defect; retest [39,40].	Diversion used as a substitute for confirmed tissue capture.
Positive air-leak test	Mechanical defect requiring localization and correction [39].	Localize; redo large, posterior, poorly visualized, or tension-related defects; repair a small viable defect; retest ± diversion.	Persistent positive test or unretested repair.
Questionable perfusion on inspection or ICG	Ischemia is not rescued by reinforcement or diversion; interpret ICG with tissue appearance, tension, and hemodynamics [22,23,54].	Release traction; optimize hemodynamics; reassess; resect to viable bowel and reconstruct if uncertainty persists.	ICG signal interpreted without tissue and hemodynamic context.
Localized staple-line concern with good perfusion and negative test	Small focal concern in viable tissue; limited evidence [58].	Targeted sutures; avoid excessive bites or luminal narrowing.	Routine circumferential oversewing.
Severe edema, contamination, conduit injury, or unsafe caliber mismatch	Multiple adverse mechanisms; poor tissue quality is not normalized by decompression or lavage [47].	Further resection/mobilization; staged reconstruction or sound anastomosis + diversion; end stoma when needed.	Unsafe reconstruction solely to avoid a stoma.

## 6. Recognition After Surgery and Diagnostic Work-Up

The diagnostic hierarchy is clinical trajectory first, biomarkers second, and imaging when concern persists. AL is often first signaled by recovery that stops progressing: fever, tachycardia, pelvic or abdominal pain, ileus, urinary symptoms, confusion, fatigue, or purulent or feculent drainage should prompt reassessment [24,27,28]. Low leaks may remain localized and diversion may blunt peritonitis, so a soft abdomen or normal drain output does not exclude pelvic sepsis. Before discharge, high-risk patients need explicit warning symptoms, rapid access to the colorectal team, and a low threshold for reassessment or CT because delay reduces salvage opportunities [27,28,29].

C-reactive protein (CRP) is most useful as a rule-out tool in an otherwise well patient, not as a stand-alone diagnosis or intervention trigger. Meta-analyses place approximate postoperative day 3–5 rule-out cutoffs within the ranges summarized in Table 3, with high negative predictive value in appropriately selected patients [49,50]. A low, falling CRP is reassuring; a persistently high or rising value should increase surveillance but remains nonspecific. Evidence for procalcitonin is less established [51].

Contrast-enhanced computed tomography (CT) is first-line imaging in stable patients with suspected AL. Rectal contrast can improve diagnostic performance when it can be administered safely and reaches the anastomosis [24,52], but a negative early CT does not exclude an intermittent or contained leak. Persistent clinical concern may therefore require repeat CT, endoscopy, examination under anesthesia, or intervention. Pelvic magnetic resonance imaging (MRI) is a problem-solving modality for equivocal CT, small defects, chronic presacral cavities, sinuses, fibrosis, or complex relationships to adjacent organs; current diagnostic estimates come from small series and do not justify replacing CT for acute first-line assessment [53,59]. Imaging should define whether contamination is localized, the cavity is drainable, it communicates with the anastomosis, and the conduit appears viable.

## 7. Management: Physiology- and Anatomy-Based Source Control

Physiology determines urgency, while anatomy determines the feasible route to source control. Hemodynamic instability, organ dysfunction, generalized peritonitis, septic shock, or uncontrolled contamination requires resuscitation, broad-spectrum antibiotics, and prompt operative control [28,29,44]. In stable patients, treatment selection should document timing, intraperitoneal or extraperitoneal location, defect size and circumference, conduit viability, diversion, and cavity drainability. Early defects in viable tissue may permit drainage, repair, or endoluminal therapy; mature fibrotic sinuses require a chronic-sepsis strategy. The goal is durable sepsis control, not preservation of an anastomosis at any cost.

### 7.1. Stable, Localized Leaks: Organ-Preserving Strategies

Small contained leaks without a drainable collection may be treated with antibiotics, temporary bowel rest, nutritional support, and close follow-up, but lack of prompt improvement should trigger repeat imaging and escalation [27,28,29]. Once a pelvic abscess or presacral cavity is present, drainage becomes central; access may be percutaneous, transgluteal, transvaginal, or transanal according to anatomy [27,28,29]. Observational pooled outcomes summarized in Table 5 suggest better apparent success with drainage than with conservative treatment alone, but this difference is heavily confounded by patient selection and should not be interpreted as head-to-head efficacy [60].

Endoluminal vacuum therapy (EVT) is the best-supported endoscopic option for a stable patient with an accessible low extraperitoneal defect, a drainable cavity, and viable tissue. Observational reviews report high cavity-healing and stoma-reversal rates, but protocols, timing, and definitions vary and no randomized comparison establishes superiority over alternative salvage strategies [61,62,63,64,65,66]. Earlier initiation may be advantageous, although small retrospective series do not define a fixed time threshold [62]. Failure is more likely with necrosis, major circumferential disruption, an inaccessible or incompletely drained cavity, ongoing fecal contamination, mature fibrosis, or persistent systemic sepsis. Clips, covered stents, and fibrin sealant have narrower indications because they do not provide the same cavity drainage; they should be used only after anatomy and source control are adequate [26,61,64,65,66].

### 7.2. Operative Source Control and Reassessment

In selected stable or stabilized patients with viable tissue, lavage, targeted drainage, proximal diversion, limited debridement, and repair of a small defect can control sepsis without dismantling the anastomosis [28,29,45]. Access may be open or laparoscopic; robot-assisted repair should remain exceptional because evidence is largely noncomparative and platform preparation must not delay treatment [42,44]. Acute preservation is not equivalent to durable healing, and failure to improve should prompt early reassessment rather than repeated preservation attempts in a septic pelvis.

Anastomotic takedown with an end stoma is appropriate when the anastomosis is nonviable or widely disrupted, diffuse contamination cannot be controlled by drainage or diversion, or a less invasive strategy has failed [60]. Redo anastomosis during acute sepsis is uncommon and should be restricted to stable patients with a healthy bowel, limited contamination, and expert teams; delayed redo reconstruction may restore continuity after chronic failure but carries substantial morbidity and uncertain function [67]. When repeated salvage is likely to prolong infection with little prospect of acceptable function, definitive diversion may be the more patient-centered endpoint.

**Figure 2:**
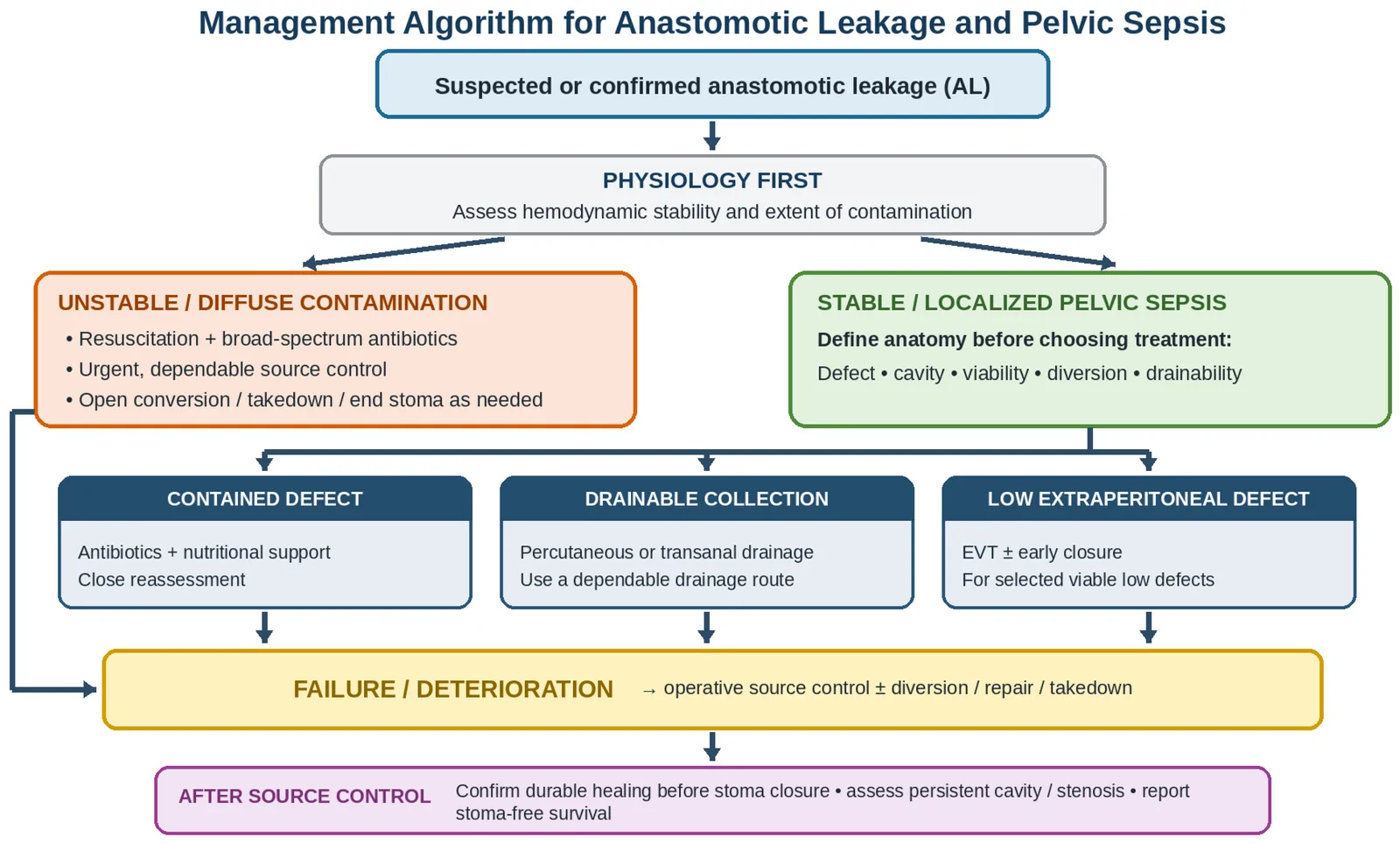
**Clinical management algorithm for AL and pelvic sepsis.** Physiological instability or diffuse contamination mandates resuscitation and prompt operative source control. In stable localized sepsis, anatomy guides treatment: selected small contained leaks without a drainable abscess may receive antibiotics and nutritional support with close reassessment (pooled success approximately 44% [60]); accessible collections may undergo percutaneous or transanal drainage (approximately 74% [60]); and selected low extraperitoneal defects in viable bowel may undergo EVT with or without early closure (EVT healing approximately 85% [61]). Clips, sutures, and glue are reserved for small viable defects after adequate drainage. Persistent fever or organ dysfunction, feculent output, an enlarging or non-collapsing cavity, or inability to advance nutrition should prompt escalation; reported pooled success is approximately 70% for salvage of a viable anastomosis [60] and approximately 77% after anastomotic takedown [60]. These pooled estimates are heterogeneous and are not head-to-head comparisons. Abbreviations: AL, anastomotic leakage; CT, computed tomography; EVT, endoluminal vacuum therapy. The colors distinguish management phases and decision points rather than quantitative risk levels.

The management evidence is substantially weaker than the prevention evidence because most salvage studies are nonrandomized, anatomically heterogeneous, and strongly affected by treatment-selection bias. Table 5 therefore summarizes the scale of reported outcomes, not a ranking of strategies [60,61,64]. Reassessment should be planned from the outset: persistent sepsis, new organ dysfunction, failure of cavity reduction, or worsening nutrition indicates inadequate source control and should trigger escalation. The clinical management algorithm is summarized in Figure 2.

## 8. Long-Term Outcomes and Patient-Centered Success

Acute sepsis control does not prove anastomotic healing. A presacral cavity may persist after drainage or diversion and evolve into a chronic sinus, with pain, intermittent inflammation, stricture, or failure of stoma reversal [10,12]. Follow-up begins with symptoms, digital examination, and endoscopy; CT or water-soluble contrast can define the cavity and luminal communication, while MRI or MRI-enema is reserved for complex sinuses, fistulas, or anatomy that remains unclear [53,59]. Established chronic sinuses may be observed, treated endoscopically or transanally, reconstructed, or managed with definitive diversion according to symptoms, frailty, cancer status, sphincter function, expected bowel function, and procedural burden [10,11,12,13,67].

Failure of reversal is a major patient-centered consequence of AL [11,13]. Before closure, systemic sepsis should have resolved, the cavity should be stable or clearly collapsing, and significant stenosis should be excluded. Digital examination and flexible endoscopy are the core tests; water-soluble contrast or CT with rectal contrast is useful after a previous leak or when an extraluminal cavity is suspected, and MRI is reserved for complex or discordant findings [53,59,68,69]. Small retrospective series suggest that selected clinically quiet sinuses may occasionally be closed before complete radiographic resolution, but this evidence is insufficient to support routine closure over a persistent cavity [69].

A more meaningful endpoint after salvage is 1-year stoma-free survival rather than avoidance of acute anastomotic takedown. In a worldwide retrospective cohort of 2470 patients with AL after rectal cancer resection, 1-year stoma-free survival varied markedly across treatment groups, but the unadjusted differences primarily reflected treatment selection and leak severity; among propensity-score-matched diverted patients, active and passive drainage showed no significant difference [13]. This finding reinforces the need to report durable continuity, time to healing and closure, secondary procedures, and treatment burden rather than acute “success” alone.

Functional recovery must be evaluated after anatomical healing. LARS can include urgency, clustering, frequency, fragmented evacuation, and incontinence, while pelvic sepsis may add fibrosis, reduced compliance, stricture, denervation, and prolonged diversion [8,9,12,14]. Management should be symptom-directed with diet, medication, pelvic-floor rehabilitation, transanal irrigation, or specialist referral [9]. For some patients, a well-functioning permanent stoma may provide better quality of life than repeated high-risk salvage followed by chronic sepsis or severe bowel dysfunction; treatment success should therefore be defined with the patient.

AL has also been associated with local recurrence and reduced survival, although residual confounding is substantial [32,33,34,35]. The immediate oncologic consequence is often delayed recovery or adjuvant treatment. Surgical and oncology teams should coordinate infection control, nutrition, surveillance, and cancer treatment. Table 6 summarizes long-term assessment and emphasizes freedom from recurrent sepsis, stoma-free survival, acceptable daily-life function, and completion of oncologic care as complementary endpoints.

## 9. Future Directions

Future prediction tools should be judged by clinical utility rather than discrimination alone. Machine-learning models using clinical or intraoperative video data remain proof-of-concept and are vulnerable to class imbalance, overfitting, center-specific technique, and poor calibration [70]. MRI radiomics and candidate biomarkers are similarly limited by few leak events, incomplete external validation, and uncertainty about whether they can change decisions such as diversion, perfusion reassessment, imaging, or reoperation [71,72]. Prospective studies should therefore report calibration, external performance, decision impact, and unintended consequences rather than simply adding another risk score.

Quantitative fluorescence is another promising but unstandardized area. A 2025 review identified 26 perfusion parameters, underscoring heterogeneity in dosing, camera distance, hemodynamic context, and analysis [73]. Future salvage studies should likewise standardize anatomy, viability, diversion, drainability, source-control failure, chronic sinus, time to closure, stoma-free survival, LARS, and quality of life. Until comparative evidence improves, disciplined intraoperative correction, early recognition, anatomy-based escalation, and planned reassessment remain more reliable than any single device or prediction model.

## 10. Conclusions

AL and pelvic sepsis remain serious after rectal cancer surgery. Open, laparoscopic, and robotic approaches create different technical conditions, but none removes the biological and mechanical vulnerability of a low pelvic anastomosis. Prevention should prioritize a well-perfused, tension-free conduit; sound transection geometry; combined bowel preparation and oral antibiotics when appropriate; and a defined response to incomplete donuts, positive leak tests, or doubtful perfusion. After surgery, clinical deterioration requires prompt reassessment, with CRP used mainly for rule-out support, CT as first-line imaging, and MRI reserved for selected complex or chronic problems. Management should be driven first by physiology and then by timing, location, defect size, conduit viability, diversion, and drainability. The most useful framework is therefore a continuous pathway linking technical prevention, recognition after discharge, anatomy-based source control, planned reassessment, and patient-centered long-term outcomes including stoma-free survival and bowel function. For minimally invasive surgery, the practical implication is not to seek a platform-specific solution to AL, but to adapt these common principles to the technical environment of laparoscopy or robotics—anticipating geometry- and traction-related failure, using adjunctive imaging selectively, and escalating or converting without delay when safe reconstruction or definitive source control cannot be achieved.

## Figures and Tables

**Table 1:** Practical risk-factor matrix for rectal anastomotic leakage.

Category	Risk Factor	Evidence Level/Quantitative Signal	Modifiability	Clinical Response
Patient	Malnutrition, hypoalbuminemia, sarcopenia	Moderate: consistent observational associations [4,5,6,7]	Modifiable/partly	Nutritional assessment and correction when oncologically appropriate; lower threshold for diversion.
	Smoking, heavy alcohol use, hyperglycemia/diabetes	Moderate: repeated cohort and review associations [4,5,6,7]	Modifiable	Cessation and metabolic optimization; avoid preventable perioperative dehydration and hypoperfusion.
	Frailty, renal, cardiopulmonary disease, high ASA status	Moderate: consistent observational associations [4,5,6,7]	Partly modifiable	Optimize physiology; consider the consequences of reoperation and stoma-related dehydration.
Anatomy	Male sex, obesity/visceral adiposity, narrow pelvis	Moderate; technically mediated [4,5,6,7]	Non-modifiable/partly	Senior involvement; plan platform, port position, stapler trajectory, and threshold for conversion.
Tumor	Low tumor requiring a very low colorectal or coloanal anastomosis	High: consistently associated with AL [4,5,6,7]	Non-modifiable	Senior review, layered prevention, selective diversion, and intensified postoperative surveillance.
	Obstruction, bulky tumor, dilated or edematous colon	Moderate: tissue quality and contamination mediated [4,5,6,7,41]	Partly modifiable	Decompress and reassess viability/caliber; avoid an unsafe low pelvic reconstruction.
Treatment	Neoadjuvant radiotherapy/chemoradiotherapy	Moderate: fibrosis and microvascular injury; effects vary [4,5,6,7]	Non-modifiable once delivered	Expect difficult dissection and impaired tissue quality; individualize diversion and follow-up.
Operative	Poor perfusion, tension, torsion, caliber mismatch	High biological plausibility with consistent clinical associations [4,5,6,7,22,23]	Modifiable	Further mobilization or resection; reconstruct if uncertainty persists; diversion does not rescue ischemia.
	Multiple rectal stapler firings	Meta-analysis: 3.5% after one vs. 6.7% after two firings; OR 2.44 [36]	Modifiable	Change stapler angle, port, transection strategy, or approach before accepting a complex staple line.
	Incomplete donuts or positive air-leak test	Moderate: a positive air-leak test identifies a mechanical defect [39], whereas an incomplete donut requires complete circumferential inspection and may warrant reconstruction [40]	Modifiable	Direct or endoscopic inspection; repair only a small localized defect in viable tissue; otherwise reconstruct the anastomosis; repeat the integrity test and consider diversion.
	Major blood loss, transfusion, contamination, prolonged difficult dissection	Moderate observational evidence [4,5,6,7]	Partly modifiable	Restore physiology; reassess reconstruction; increase threshold for diversion or abandonment.
System	Learning curve, low team familiarity, delayed recognition after discharge	Limited-to-moderate: process- and experience-dependent [15,16,17,18,19,20,21,24,25,42]	Modifiable	Standardized team workflow, senior support, direct post-discharge contact, and early reassessment.

**Table 2:** Approach-specific technical factors that may influence anastomotic leakage and their preventive implications.

Approach	Technical Characteristics	Potential Mechanisms Affecting AL	Preventive Implications
Open	Palpation; tactile assessment; flexible instrument exchange.	Limited deep-pelvic exposure; difficult low suturing; retraction-related tissue trauma.	Optimize exposure; verify reach, perfusion, donuts, and leak test; change approach if access is inadequate.
Laparoscopic	Magnification; established workflow; ICG fluorescence; no docking.	Restricted stapler angles; tissue torque; oblique transection; multiple firings.	Plan ports/stapler entry; minimize firings and traction; mobilize fully; alter approach if alignment is unsafe.
Robotic	3D view; wristed instruments; tremor filtration; controlled retraction; ICG fluorescence.	No tactile feedback; visually estimated traction; docking/arm/stapler constraints; learning curve.	Standardize traction/stapling/docking; use articulation for exposure; do not accept ischemia, tension, or unsound tissue.

**Table 5:** Treatment pathways and reported outcomes for rectal anastomotic leakage and pelvic sepsis.

Strategy	Typical Selection	Reported Success/Healing	Long-Term Considerations
Conservative management	Stable; small contained leak; no drainable abscess; rapid clinical improvement.	44.4% pooled success [60].	Risk of delayed source control; pooled long-term stoma rate 33.3% [60].
Percutaneous/ transanal drainage	Stable; localized, accessible abscess or presacral cavity.	73.7% pooled success [60].	Reintervention may be required; pooled long-term stoma rate 22.5% [60].
EVT ± early closure	Stable; accessible low extraperitoneal cavity; viable tissue; repeated endoscopy feasible.	85.3% cavity healing; 75.9% stoma reversal in observational review [61].	Protocol burden; stricture/sinus; outcomes depend on timing, anatomy, and diversion.
Anastomosis-conserving surgery	Stable or stabilized; viable anastomosis; contamination controllable by lavage/drainage/diversion ± repair.	69.7% pooled success [60].	Pooled long-term stoma rate 27.6%; acute preservation may not equal durable continuity [60].
Anastomotic takedown	Necrosis, major dehiscence, diffuse contamination, or failed salvage.	77.0% pooled success [60].	Pooled long-term stoma rate 46.0%; often reflects greater initial severity [60].

**Table 6:** Long-term consequences and assessment after anastomotic leakage and pelvic sepsis.

Issue	Assessment	Clinical Implication	Patient-Centered Endpoint
Chronic presacral sinus	Symptoms, endoscopy, CT or contrast study; MRI/MRI-enema for complex sinus or fistula.	Do not rush stoma closure; define whether the cavity is closing or fibrotic.	Freedom from recurrent pelvic sepsis.
Delayed/failed reversal	Endoscopy; water-soluble contrast study or CT with rectal contrast after a previous leak; MRI/MRI-enema for complex sinus or discordant findings [53,59,68,69].	Do not rush closure with active sepsis, a non-collapsing cavity, or important stenosis. Selected short, clinically quiet sinuses may be considered only after concordant assessment [69].	Stoma-free survival and time to closure.
Bowel dysfunction	LARS score and detailed functional history.	Treat function after closure, not anatomy alone.	Acceptable daily-life function and continence.
Quality of life	Patient-reported symptoms, treatment burden, goals.	Balance salvage against repeated procedures and poor function.	Goal-concordant recovery.
Oncologic trajectory	Delay in adjuvant therapy and surveillance.	Coordinate infection recovery with cancer treatment.	Timely completion of oncologic care.

## Data Availability

No new data were created or analyzed in this study. Data sharing does not apply to this article.
